# Development and characterisation of SMURF2-targeting modifiers

**DOI:** 10.1080/14756366.2020.1871337

**Published:** 2021-01-12

**Authors:** Dhanoop Manikoth Ayyathan, Gal Levy-Cohen, Moran Shubely, Sandy Boutros-Suleiman, Veronica Lepechkin-Zilbermintz, Michael Shokhen, Amnon Albeck, Arie Gruzman, Michael Blank

**Affiliations:** a Laboratory of Molecular and Cellular Cancer Biology, Azrieli Faculty of Medicine, Bar-Ilan University, Safed, Israel; b Department of Chemistry, Bar-Ilan University, Ramat Gan, Israel

**Keywords:** SMURF2, autoubiquitination, peptides, peptidomimetics

## Abstract

The C2-WW-HECT-domain E3 ubiquitin ligase SMURF2 emerges as an important regulator of diverse cellular processes. To date, SMURF2-specific modulators were not developed. Here, we generated and investigated a set of SMURF2-targeting synthetic peptides and peptidomimetics designed to stimulate SMURF2’s autoubiquitination and turnover via a disruption of the inhibitory intramolecular interaction between its C2 and HECT domains. The results revealed the effects of these molecules both *in vitro* and *in cellulo* at the nanomolar concentration range. Moreover, the data showed that targeting of SMURF2 with either these modifiers or *SMURF2*-specific shRNAs could accelerate cell growth in a cell-context-dependent manner. Intriguingly, a concomitant cell treatment with a selected SMURF2-targeting compound and the DNA-damaging drug etoposide markedly increased the cytotoxicity produced by this drug in growing cells. Altogether, these findings demonstrate that SMURF2 can be druggable through its self-destructive autoubiquitination, and inactivation of SMURF2 might be used to affect cell sensitivity to certain anticancer drugs.

## 1. Introduction

Ubiquitin-based protein modification, mediated by the concerted action of ubiquitin-activating factors (E1s), ubiquitin-conjugating enzymes (E2s), and E3 ligases (E3s), controls a plethora of essential molecular and cellular processes both through proteolytic and non-proteolytic mechanisms. These include the regulation of protein turnover and localisation, protein–protein interactions and signal transduction, DNA replication, transcription, damage repair, immune responses, and cell death. Target-oriented E3 ligases provide the specificity to ubiquitin-mediated signalling. It is therefore not surprising that E3s are under intensive investigation as disease biomarkers and drug targets in different pathobiological settings.

Smad ubiquitination regulatory factor 2 (SMURF2) is a member of the HECT-type NEDD4 E3 ligase family. This family is characterised by the presence in the protein structure of the C2 domain (a calcium and lipid binding domain of ∼120 amino acids), several tryptophan-rich WW domains (∼40 residues long; play a role in protein–protein interactions via recognition of proline‐rich motifs in target proteins), and the catalytical carboxy-terminal HECT domain (∼350 residues). HECT domain consists of N- and C-terminal lobes connected through a flexible hinge allowing them to come together during ubiquitin transfer. The N-lobe interacts with E2, whereas the C-lobe harbours the active-site cysteine (Cys716 in SMURF2) forming the thioester bond with ubiquitin. Despite high similarities in the domain composition, NEDD4 E3s have distinct substrate repertoire and reveal distinct roles in physiological and pathobiological processes, including cancer^1–4^. In neoplastic diseases, SMURF2 was shown to exert both tumour-promoting and suppressor activities, depending on tumour type, stage, molecular and cellular contexts, and other still unidentified factors^5^.

SMURF2 was shown to ubiquitinate and regulate stability, localisation, and functions of several critical proteins pertinent to cancer initiation, progression, and therapeutic response. These include TGF-β receptor and SMAD transducers^6–9^, the components of the Wnt/β-catenin signalling pathway Axin^10^ and GSK3β^11^, DNA topology regulator Topo IIα^12^, epigenetic modifiers RNF20^13^^,^^14^, EZH2^14–16^, SIRT1^17^, transcription factors KLF5^18^, YY1^19^, SATB1^20^, ChREBP^21^, nuclear lamins^22^^,^^23^, as well as HECT- and RING-type E3s^24–26^. This broad target repertoire of SMURF2 suggests that modulation of its protein abundance and activity could have a profound impact on several essential molecular and cellular processes involved in the disease onset, progression, and therapeutic response. However, despite these proceedings SMURF2-targeting agents have not yet been developed.

Previous studies showed that the catalytic activity of SMURF2 is tightly regulated by its intramolecular interactions, particularly between its C2 and HECT domains. It has also been shown that the C2 domain of SMURF2 binds to the HECT domain in a close vicinity to the active-site cysteine, inhibiting E2–E3 trans-thiolation and ubiquitin-thioester bond formation^27^, thereby impeding the ability of SMURF2 to ubiquitinate its protein substrates including SMURF2 itself. Based on these findings, we hypothesised that disruption of this regulatory mechanism by small molecules would initially activate SMURF2, yet consequently diminish its cellular levels due to its autoubiquitination and turnover. To experimentally test this hypothesis, we generated and investigated a set of SMURF2-targeting short peptides and peptidomimetics designed to interfere with the complex formation between the SMURF2’s C2 domain and HECT domain. Molecular dynamics (MD) simulations were carried out to identify the interactions between HECT, C2, and small molecule inhibitors. The ability of these compounds to affect SMURF2 autoubiquitination and cellular abundance, as well as their effects on cell growth and sensitivity to the genotoxic drug etoposide, were analysed *in vitro* and *in cellulo*.

## 2. Materials and methods

### 2.1. Peptide synthesis and purification

#### 2.1.1. General remarks

Peptides were synthesised on two different resins: Rink Amide and HMBA-AM (applied Chem-Impex). Anhydrous DMF was obtained by distillation under vacuum and stored over 4 Å molecular sieves. The peptides were purified by preparative reversed-phase HPLC. Analytical and preparative HPLC were performed on LUNA C18 preparative (10 µm, 100 × 30 mm) or analytical (5 µm, 250 × 4.6 mm) columns, both from Phenomenex, Inc. (Torrance, CA). HPLC purification was carried out with an increasing linear gradient of CH_3_CN in H_2_O. Mass spectra were recorded on a QToF microspectrometer, using electrospray ionisation (ESI) in the positive/negative ion mode. Data were processed using mass L-ynX ver. 4.1 calculation and de-convolution software (Waters Corp., Milford, MA). HRMS were obtained using an LTQ Orbitrap.

#### 2.1.2. General procedure for the synthesis on HMBA-AM resin

Coupling of the first Fmoc (9-fluorenylmethoxycarbonyl)-protected amino acid was achieved with N,N′-diisopropylcarbodiimide (DIC), 4-dimethylaminopyridine (DMAP) in dry DMF for 4 h. The remaining Fmoc-protected amino acids were coupled with O-(benzotriazol-1-yl)-N,N,N′,N′-tetramethyluronium (HBTU) in DMF in combination with N,N-diisopropylethylamine (DIPEA), for a 1.5 h cycle. Fmoc deprotection was achieved with piperidine. Side-chain deprotection was achieved by treating the peptide with 5 ml of 95% trifluoroacetic acid (TFA), 2.5% triisopropylsilane (TIS), and 2.5% H_2_O. Peptide cleavage from the resin was achieved by treating the resin-bound peptides with 4 ml of 1:3 1 M NaOH:dioxane and then by 1 M HCl. The peptides were lyophilised and purified by preparative reversed-phase HPLC. The molecular mass of the peptides was determined by either MALDI-TOF or ESI mass spectrometry. The peptides that were synthesised on the HMBA-AM resin are listed below.

Pep1 – LR(Pbf)FF: ESI (*m/z*) [MH]^+^ 834.4; [MNa]^+^ 856.4.
Pep2 – D(OBtu)PLR(Pbf)FF: ESI (*m/z*) [MH]^+^ 1102.5; [MNa]^+^ 1124.5; [MK]^+^ 1140.5.
Pep3 – DPLRFF: ESI (*m/z*) [MH]^+^ 794.4; [MNa]^+^ 816.5; [MK]^+^ 832.5.
Pep4 – PLR(Pbf)FFD(OBtu): ESI (*m/z*) [MH]^+^ 1102.4; [MNa]^+^ 1124.4; [MK]^+^ 1140.0.
Pep5 – FFRLPD: ESI (*m/z*) [MH]^+^ 794.4.

#### 2.1.3. General procedure for the synthesis on Rink amide resin

Coupling of Fmoc-protected amino acids was achieved by using HBTU in DMF in combination with DIPEA, for a 1 h cycle. Fmoc deprotection was conducted with piperidine. N-terminus acetylation was carried out by using 10 ml of the acetylation solution, which contains 19 ml of acetic anhydride, 9 ml DIPEA, 6 mmol of HOBt, and 72 ml of NMP (N-methyl-2-pyrrolidone), for 35 min. This procedure was carried out twice. A 6-(Fmoc-amino)hexanoic acid (4 eq), which in some peptides were used as a linker, was coupled under HOBt (3 eq), Bop (3 eq), and DIPEA (6 eq) treatment in DMF for 1.5 h. Side-chain deprotection and peptide cleavage from the resin were carried out by treating the resin-bound peptides with 5 ml of 95% TFA, 2.5% TIS, and 2.5% H_2_O. The peptides were washed three times with cold diethyl ether, vortexed, and then centrifuged for 5 min at 3500 rpm. The peptides were then purified by preparative reversed phase HPLC. The mass of the peptide was confirmed either by MALDI-TOF MS or by ESI mass spectrometry. The peptides that were synthesised using Rink amide resin are listed below.

Pep6 – Ac-LRFF-NH_2_: ESI (*m/z*) [MH]^+^ 623; [MNa]^+^ 645^;^ [MK]^+^ 661.
Pep7 – Ac-LRFFDK-NH_2_: ESI (*m/z*) [MH]^+^ 866.4.
Pep8 – Ac-DFFRLP-NH_2_: ESI (*m/z*) [MH]^+^ 835.6.
Pep9 – Ac-FFRL-NH_2_: ESI (*m/z*) [MH]^+^ 623.4.
Pep10 – Ac-KDFFRL-NH_2_: [MH_2_]^2+^ 433.8; [MH]^+^ 866.4.
Pep11 – Ac-FFRLPD-NH_2_: ESI (*m/z*) [MH]^+^ 835.6.
Pep12 – Ac-PLR-NaI-D-NH_2_: ESI (*m/z*) [MH]^+^ 738.4.
Pep13 – Ac-PLRFAD-NH_2_: ESI (*m/z*) [MH]^+^ 759.4.
Pep14 – Ac-PLRAFD-NH_2_: ESI (*m/z*) [MH]^+^ 759.4; [MNa]^+^ 781.4.
Pep15 – Ac-HKKIHKK-NH_2_: ESI (*m/z*) [MH_3_]^3+^ 320.7; [MH_2_]^2+^ 480.5; [MH]^+^ 959.7.
Pep16 – Ac-NTLDPK-NH_2_: ESI (*m/z*) [MH]^+^ 728.5.
Pep17 – Ac-PLAPYD-NH_2_: ESI (*m/z*) [MH]^+^ 851.4.
Pep18 – Tetrzaol-FFRLP-NH_2_: ESI (*m/z*) [MH]^+^ 788.3; [MNa]^+^ 810.4.
Pep19 – Ac-LRAA-NH_2_: ESI (*m/z*) [MH]^+^ 471.

### 2.2. Computational protocol

#### 2.2.1. Generation of preliminary 3D structures of molecular systems: HECT–C2 and HECT–peptide inhibitor complexes

3D structures of the separated C2 and HECT domains of SMURF2 were acquired from the solution structure of C2 2JQZ.pdb^27^ and the crystal structure of the HECT fragment 1ZVD.pdb^28^. Initial structure of the HECT–C2 complex was generated by means of the Discovery Studio 4.0 molecular modelling package^29^, implementing methods ZDOCK^30^, and RDOCK^31^ for rigid body protein docking. The docked poses generated by ZDOCK were filtered by a set of C2 residues experiencing the most significant NMR chemical shift in the complex with HECT^27^. On the next step, RDOCK protocol was used, providing optimisation of docked poses generated by the ZDOCK protocol. RDOCK consists mainly of a two-stage energy minimisation scheme that includes the evaluation of electrostatic and desolvation energies. During the two-stage energy minimisation, RDOCK takes advantage of CHARMM molecular modelling software^32^ to remove clashes and optimise polar and charge interactions. Finally, the best docking pose was used as input structure for the subsequent MD simulation. Pep3, Pep5, Pep7, and Pep10 designed, synthesised and experimentally estimated in this work as promising inhibitors of SMURF2, were used for the construction of 3D structures of their complexes with HECT by molecular modelling. The best poses of the HECT-peptidyl inhibitor complexes generated by VINA docking algorithm^33^, implemented in the YASARA structure software^34^, were used as initial 3D structures for the subsequent MD simulations.

#### 2.2.2. Construction of systems for MD simulations

Standard AMBER molecular dynamics software^35^ procedures were used for the generation of input structural and topological files for all target systems under MD simulations in frames of the FF14SB force field (for protein, peptide substrates, and solvent molecules)^36^. The generated script files controlling all steps of simulations at 310 K used the 10 Å cut-off. The solute (protein system) was placed in the solvent box with minimal distance of 15 Å to its borders. Solvent water molecules as TIP3P model were filled into the box. The electrostatic charge of the simulated systems was neutralised by addition of Na^+^ or Cl^–^ counter ions. The MD simulated HECT–C2 complex consists of 373 (HECT) and 131 (C2) amino acid residues, 34,219 solvent water molecules, and nine Cl^–^ counter anions, constitute 111,219 atoms in total. Both protein fragments of SMURF2, HECT^28^, and C2^27^, as acquired from the Protein Data Bank, were protected by capping groups at their N- and C-ends by Ac and NMe, respectively. Standard protonation states were used for all ionisable residues: negatively charged Asp, Glu, and positively charged Arg and Lys residues. All His residues where neutral, containing one proton on Nδ except for His 484, 530, and 714 of HECT with one proton on Nε. The Na^+^ cation was deleted from HECT structure since it was artificially added in the crystallisation procedure^28^.

#### 2.2.3. *MD simulations by AMBER16 software* package

##### 2.2.3.1. Minimisation

The first stage was three sequential steps of energy minimisation:

1. Minimise only the water, restraining the protein (20,000 cycles).
2. Let water move (NTP, 300 K), restraining the protein.
3. Unrestrained minimisation of all system – water and protein (20,000 cycles).

##### 2.2.3.2. Heating

After the initial minimisation, the system was slowly heated in 1 ns from 0 K to the production temperature of 310 K. The Langevin thermostat was used. The SHAKE constraints were used to fix hydrogen atom bond lengths allowing to run with a 2 fs time step. Since in low temperature the calculation of pressure is inaccurate, the response of the barostat can distort the system, so MD simulation was conducted in NVT ensemble. The protein molecule was restrained using harmonic approximation with force constant 10 (kcal/mol)/Å^2^.

##### 2.2.3.3. MD equilibration

After the system was heated to 310 K, allowing the density of the system to equilibrate, we ran 10 repeated MD restarts each of 500 ps with a time step of 2 fs (SHAKE constraint) under Langevin thermostat and NPT ensemble with pressure of 1 atm. No positional restraints were applied. Random seeds by pseudorandom number generator were used to restart the simulations in repeated segments.

##### 2.2.3.4. Production MD stage

In the previous equilibration stage, the temperature of 310 K and stable density were reached. In this last stage, we ran production dynamics with Langevin thermostat and NPT ensemble under pressure of 1 atm and SHAKE constraint of 2 fs. The total run time is varied for each molecular system depending on its RMSD convergence. In all systems, the MD production simulation started from two 50 ns sequential steps followed by a set of 100 ns steps. Every MD step was restarted from the previous one applying random seeds generator.

#### 2.2.4. Post processing analysis

##### 2.2.4.1. Conformational cluster analysis

By means of CPPTRAJ^37^^,^^38^, implemented in AMBER 16 package, we analysed the RMSD convergence of the MD trajectories, and provided cluster analysis of protein conformations on the equilibrated final fragment of the trajectories in order to identify 3D structure of protein systems corresponding to cluster centroids.

##### 2.2.4.2. Calculation of inhibitors binding energies

Implemented in AMBER, the MM-PBSA.py method^39^ was applied for calculation of free energies of binding of peptide inhibitors to HECT in frames of the generalised Born model^40^. The values of free energies of binding of HECT with peptide inhibitors were calculated on previously generated production MD trajectories. The MD trajectory fragment of 2 ns containing 200 frames centred around the corresponding conformational cluster centroid were used in every simulated HECT-inhibitor complex for the calculation of inhibitor averaged free binding energy. The entropy calculation by performing normal mode analysis was ignored as computationally too expensive and because of a potential source of the results uncertainty.

### 2.3. Biological experiments

#### 2.3.1. Cell cultures and reagents

ANJOU-65 cells were propagated in RPMI-1640 medium supplemented with 2 mmol/l l-glutamine (Gibco, Carlsbad, CA), 10% (v/v) foetal bovine serum (FBS), and 1% (v/v) Pen-Strep (Biological Industries, Bet-Haemek, Israel). DU-145 and MDA-MB231 cells were cultured in high glucose DMEM (4.5 g/l d-glucose, Gibco, Carlsbad, CA) supplemented with 10% (v/v) FBS serum, 2 mmol/l l-glutamine and 1% (v/v) Pen-Strep. All cells were incubated at 37 °C in 5% CO_2_. Cell authentication was performed at the Genomic Center of Biomedical Core Facility (Technion, Haifa, Israel). Etoposide was obtained from Calbiochem (San Diego, CA), XTT and trypan blue cell viability reagents were purchased from Biological Industries (Bet-Haemek, Israel).

#### 2.3.2. Generation of SMURF2-depleted cells

To generate *SMURF2* knock-down, cells were infected with lentiviral particles containing one of the following shRNAs: shSMURF2#1 (targets *SMURF2* at its coding sequence; Cat. #TRCN0000010792, Sigma, St. Louis, MO) and shSMURF2#2 (targets *SMURF2* at 3′UTR; Cat. #TRCN0000003475, Sigma, St. Louis, MO). shRNA targeting luciferase (shLuc; Cat. #SHC007, Sigma, St. Louis, MO) served as a control^12^. Following infection, cells were selected with puromycin (1–2 µg/ml) for at least two weeks. SMURF2 knock-out cells were generated using an advanced CRISPR/Cas9 gene-editing tool, as previously described^41^. The efficiency of *SMURF2* depletion was verified in immunoblots.

#### 2.3.3. Whole protein extracts and immunoblot analysis

Protein extraction and western blot analyses were conducted as previously described^12^. Briefly, cells were lysed in RIPA buffer (50 mM Tris–HCl (pH 7.8), 1% NP40 substitute, 150 mM NaCl, 0.1% SDS, 0.5% sodium deoxycholate), supplemented with protease (Roche, Basel, Switzerland) and phosphatase inhibitors (Sigma, St. Louis, MO). The samples were then incubated on ice for 30 min and sonicated for 1 min at 30% amplitude. After sonication, the samples were centrifuged (10 min at 14,000 rpm), supernatants collected, and their protein concentrations determined using Pierce BCA protein assay kit (Thermo Scientific, Waltham, MA). Protein extracts were resolved in SDS-PAGE and detected in immunoblots using the following primary antibodies: anti-SMURF2 (Cat. #12024, 1:1500; Cell Signaling, Boston, MA), anti-β-actin (Cat. #600401886, 1:3000, Rockland, Pottstown, PA), or anti-α-tubulin (Cat. #T9026, 1:5000; Sigma, St. Louis, MO). The corresponding secondary horseradish peroxidase-conjugated antibody (Cat. #711-036-152 and #711-036-151, Jackson ImmunoResearch Laboratories, West Grove, PA) was used at the dilution 1:10,000. The membranes were then developed using a chemiluminescent substrate (WesternBright ECL HRP substrate, Cat. # K-12045-D20, Advansta, San Jose, CA) and visualised in SyngeneG:BOX (Syngene, Copenhagen, Denmark). Quantification of the data from the blots was performed using Gel.Quant.NET.

#### 2.3.4. Protein production and SMURF2 autoubiquitination assay

GST and GST-SMURF2 fusion proteins, including SMURF2 wild-type (SMURF2WT) and its catalytically inactive mutant form (Cys716Ala, SMURF2*Mut*) were produced and purified from *E. coli* using glutathione sepharose 4B beads (GE Healthcare, Chicago, IL)^12^. SMURF2 autoubiquitination assays were conducted with or without the selected SMURF2-targeting peptides in the presence of 250 ng of GST-SMURF2 (or GST as a control), 5 μg HA-ubiquitin (Cat. #U-110, Boston Biochem, Cambridge, MA), 100 ng E1 (UBE1, Cat. #E-305, Boston Biochem, Cambridge, MA), 150 ng E2 enzyme (UbcH5c/UBE2D3, Cat. #E2-627, Boston Biochem, Cambridge, MA), and 1 mM ATP-Mg (Cat. #B-20, Boston Biochem, Cambridge, MA) in the 1X ubiquitin conjugation reaction buffer (Cat. #B-70, Boston Biochem, Cambridge, MA), for 2 h at 37 °C. The reactions were stopped with RIPA buffer and GST proteins were pull-down using glutathione sepharose beads. Subsequently, the samples were washed four times with ice-cold RIPA buffer and eluted from the beads using 5× SDS gel-loading buffer (50 mM Tris–HCl (pH 8), 5 mM EDTA, 5% SDS, 50% glycerol, 50 mM DTT, 0.05% w/v bromophenol blue, 6% β-mercaptoethanol). Following SDS-PAGE, the autoubiquitination of SMURF2 was analysed in immunoblots using anti-HA antibody (Cat. #715500, Thermo Scientific, Waltham, MA).

#### 2.3.5. Cell proliferation and viability assays

Cell proliferation and viability were measured using the XTT and trypan blue exclusion assays, as previously described^14^. Briefly, for the XTT assay, cells were seeded at equal density (3 × 10^3^ cells/well) in 96-well plates. SMURF2-targeting molecules and/or etoposide were administered to the cells 24 h later. After 72 h, XTT reagent was added and plates were incubated for additional 5 h. The absorbance was then measured using Eon Microplate Spectrophotometer (BioTek, Winooski, VT) at 475 nm, with a reference wavelength set on 660 nm. For the trypan blue exclusion assay, cells were plated in 60 mm dishes at the density of 10^5^ cells/plate, and allowed to grow for the indicated period of time. Subsequently, cells and their supernatants were collected and, following staining with 0.2% trypan blue, analysed in Cellometer^TM^ Auto T4 Cell Counter (Nexcelom Bioscience, Lawrence, MA).

#### 2.3.6. Statistical analysis

Two-tailed Student’s *t*-test was used for data analysis. *p* Values less than 0.05 were considered statistically significant.

## 3. Results and discussion

### 3.1. Design and synthesis of SMURF2-targeting peptides and peptidomimetics

**Figure 1.**
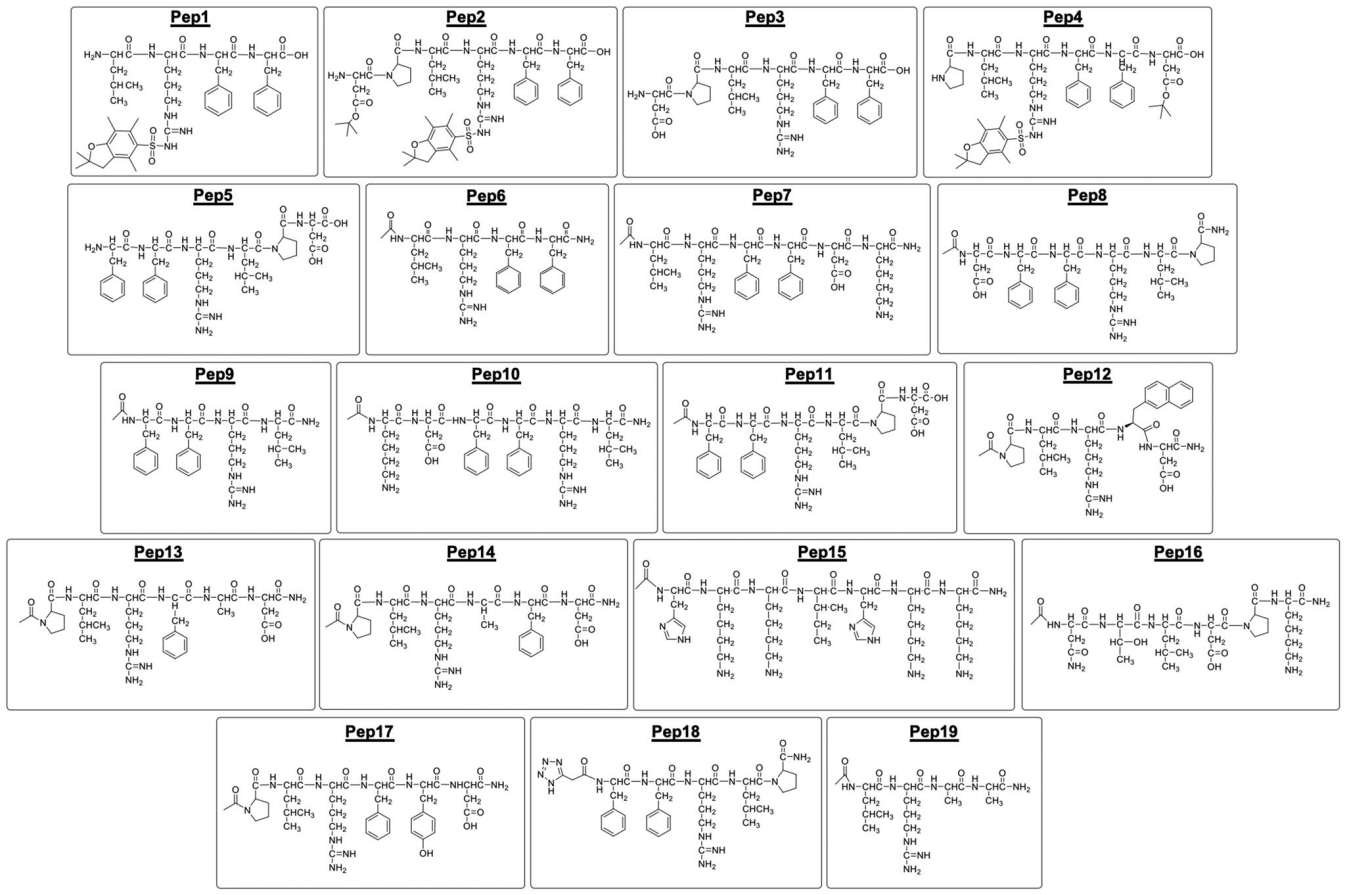
2D structural formulas of SMURF2-targeting peptides and peptidomimetics, synthesised and investigated in this study.

Based on 2D NMR, it was shown that Phe29, Phe30, and Arg31 in SMURF2’s C2 domain and hydrophobic/acidic residues surrounding Ile402 and Ile489 in its HECT domain act as reciprocal binding sites required for the C2–HECT intramolecular interaction. This interaction enables SMURF2 autoinhibition^27^. Based on these data, we designed and synthesised 19 short compounds (peptides and peptidomimetics) located either proximal or distal to the Phe29-Phe30-Arg31 site (Figure 1). Some of these compounds (i.e. Pep1, Pep2, Pep3, Pep4, Pep6, Pep7, Pep11, Pep14, and Pep19) were synthesised in the reverse order: Arg31-Phe30-Phe29. Additionally, in certain peptides (i.e. Pep1, Pep2, and Pep4), the protecting groups of side chains were left uncleaved in order to increase the chemical space diversity. Finally, in compound Pep19, two phenylalanine residues were replaced with alanines; whereas in Pep12 and Pep18 compounds we incorporated non-physiological amino acids. Fourteen out of 19 compounds were synthesised by on Rink amide resin (Pep6, Pep7, Pep8, Pep9, Pep10, Pep11, Pep12, Pep13, Pep14, Pep15, Pep16, Pep17, Pep18, and Pep19), while the others were generated using HMBA-AM resin (Pep1, Pep2, Pep3, Pep4, and Pep5), as detailed in the materials and methods.

### 3.2. Molecular modelling of the selected SMURF2-targeting molecules in complex with the HECT and HECT–C2 domains and structural analysis of the results

**Figure 2.**
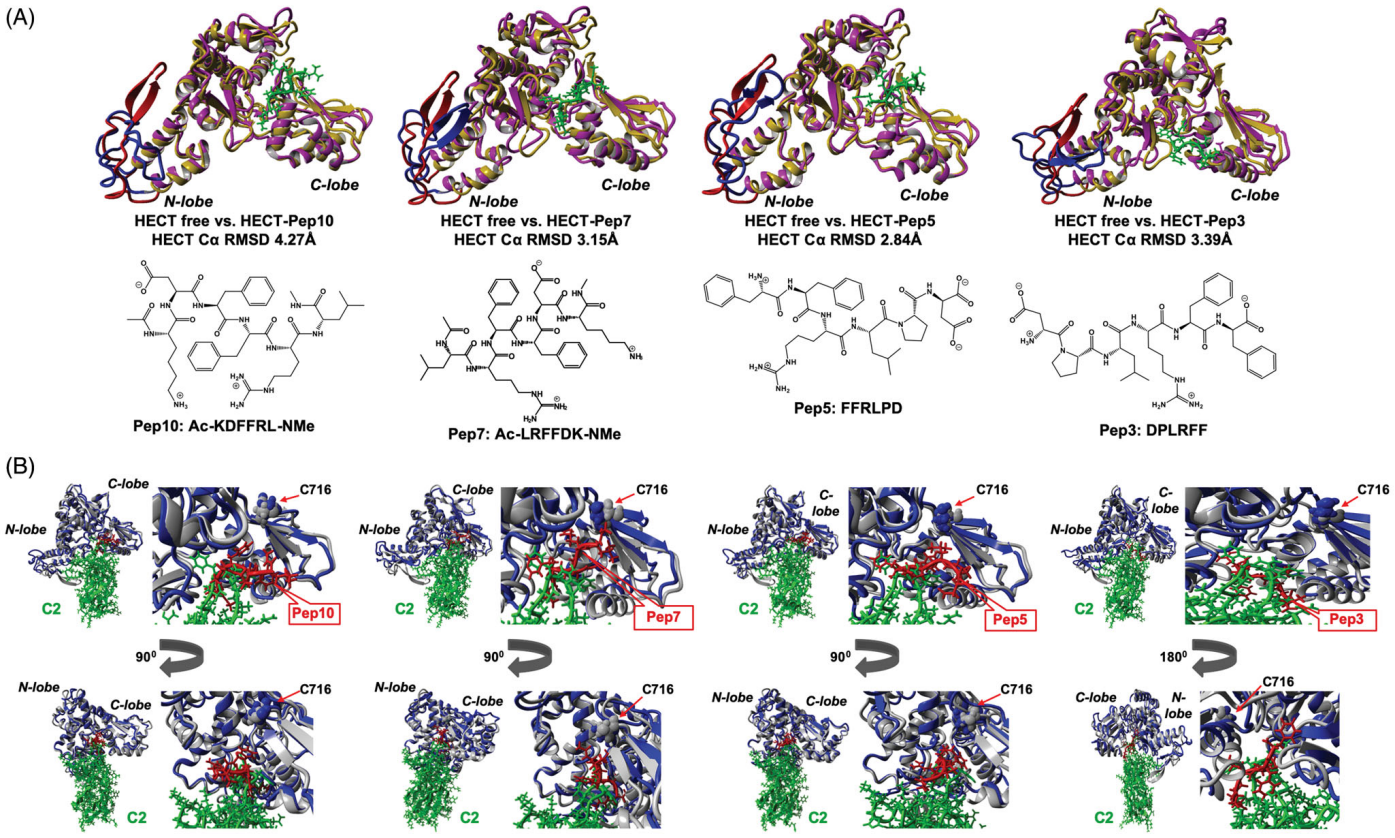
Comparative structural analysis of the following 3D structures: SMURF2–peptide complexes, HECT–C2 complex, and free HECT domain of SMURF2. The analysed molecular structures are the conformational cluster centroids (see details in the main text). Colour scheme: HECT free – orange, HECT in complex – magenta, peptide inhibitor – green. The HECT N-lobe Glu538–Lys576 fragment constituting the E2 binding site is coloured in red and blue for the free and bound forms of HECT, respectively. (A) Superposition of free HECT domain with HECT–peptide complexes. 2D structures of peptide inhibitors. (B) Superposition of HECT–C2 complex with HECT–peptide inhibitors complexes in two projections. Left images show the general view. The magnifying views on the inhibitors binding sites are shown on the right.

We generated by molecular modelling the 3D structures of HECT–C2 complex and four HECT complexes formed with two selected pairs of peptide inhibitors: Pep5 and Pep10 and their counterparts with the reverse amino-acids sequences: Pep3 and Pep7, respectively. These peptides are all of six amino acids long and include the Phe29-Phe30-Arg31 residues needed for C2 binding to HECT. They also showed the significant biological effect in *in cellulo* studies (described below). The visual molecular modelling by Yasara structure software was the first step for the generation of 3D structures of the peptide inhibitors in complex with the HECT and C2 domains. The protein–protein docking algorithm was applied for the construction of initial structure of the HECT–C2 complex. The HECT–peptide inhibitor complexes were also generated by the flexible docking method^30^^,^^31^. By applying the AMBER16 software package^35^, the initial structures of free HECT and its complexes with selected inhibitors were then corrected by means of sequential steps procedure, starting from a geometry optimisation followed by MD simulations. We have identified the 3D structures of conformational cluster centroids corresponding to the most stable conformational states of free HECT and its complexes with C2 and the small peptides (Supplementary Figure S1A). The cluster analysis was provided on the relaxed end-fragments of production MD trajectories controlled by RMSD of protein backbone Cα atoms (Supplementary Figure S1B). The results revealed that all tested molecules are bound to the HECT fragment locating between the N- and C-lobes of HECT and could serve as competitive inhibitors of SMURF2’s HECT conformational flap required for the rapprochement of the ubiquitin-loaded E2 with the active-site of SMURF2. Moreover, the results pointed out that all tested compounds have allosteric influence on the HECT N-lobe Glu538–Lys576 fragment constituting the E2 binding site (Figure 2(A)). The mentioned small molecule peptide inhibitors compete also with C2 in its binding to HECT as an intrinsic SMURF2 factor regulating catalytic activity of HECT E3 ubiquitin ligase (Figure 2(B)).

Using the MM-PBSA algorithm^39^ in frames of the generalised Born method^40^, we also calculated free energies of binding and derived by standard thermodynamic equation corresponding dissociation constants (*K*_D_) of C2 and peptide inhibitors to the HECT domain (Supplementary Figure S1C). We should note that the generalised Born method usually considerably overestimates the absolute values of free energies of binding and, consequently, the dissociation constants, so only a trend in the relative free energy of binding and *K*_D_ values could serve for the comparison between calculated and any experimental estimations of the considered inhibitor binding affinity. Comparison of the calculated trend in free energies of binding of C2 with the four small peptides predicts that the HECT–C2 complex is much more stable than HECT complexes with the small non-covalent peptide inhibitors. The latter correlates with the fact that the binding affinities of protein non-covalent inhibitors are far superior to small molecule non-covalent inhibitors of the same enzyme. The calculated free energies of binding predict that HECT complex with Pep10 is more stable than with Pep5, and Pep7 is stronger inhibitor than Pep3. Moreover, the inhibitors with the reverse sequence, Pep3 and Pep7, form more stable complexes with HECT than their counterparts Pep5 and Pep10 with the original sequence acquired from C2, correspondingly. The relevance of the provided inhibitors binding trend prediction is supported by the experimental findings presented in the next sections.

### 3.3. Pep3 and Pep7 facilitate the autoubiquitination of SMURF2

**Figure 3.**
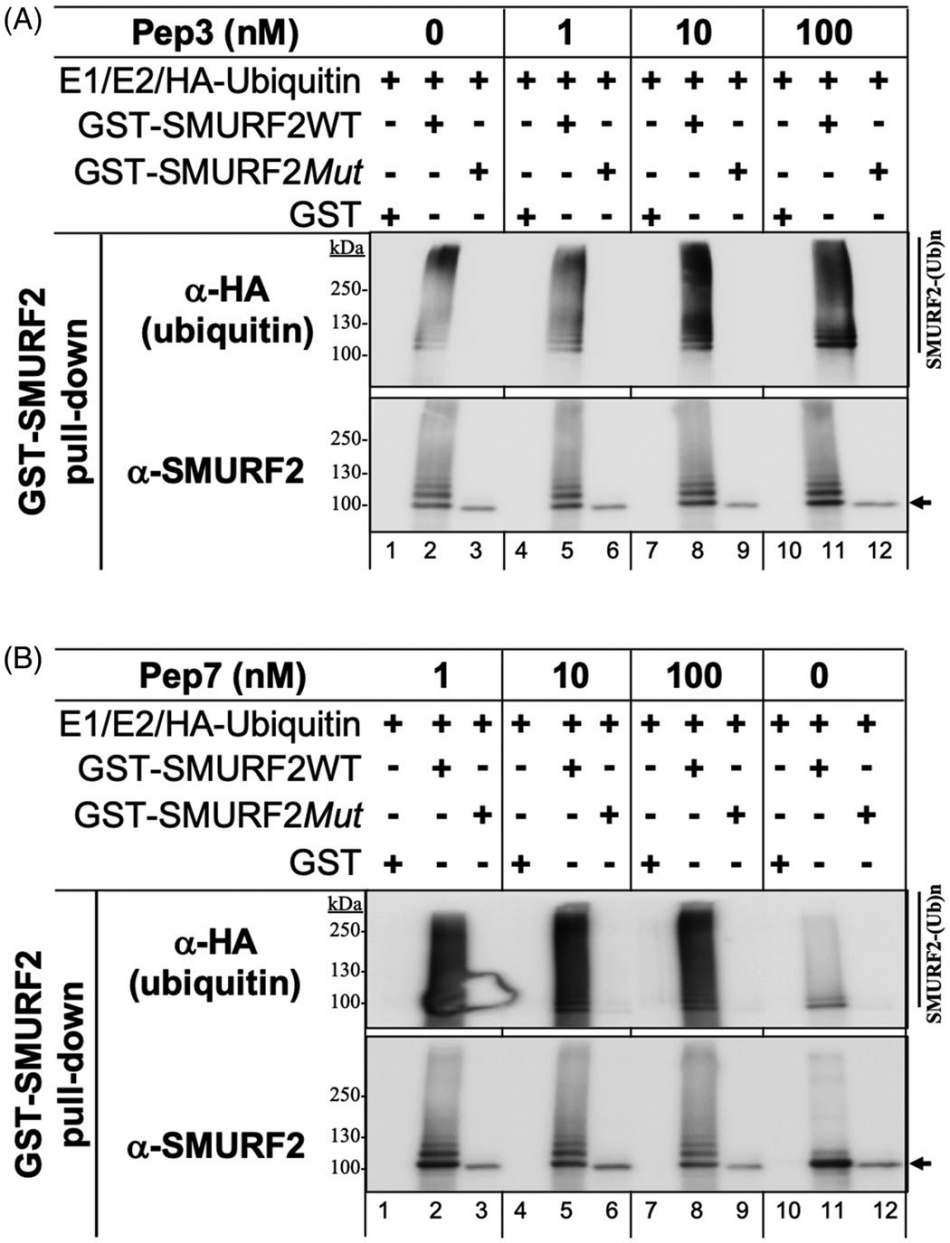
Pep3 and Pep7 increase SMURF2 auto-ubiquitination at the nanomolar range. The ubiquitination reconstitution reactions with purified recombinant GST-SMURF2 and other components of the ubiquitination cascade were performed in the tube with or without the indicated peptides (at the concentrations ranged 1–100 nM). SMURF2 (wild-type, GST-SMURF2WT, or its catalytically-deficient C716A form, GST-SMURF2*Mut*) were pulled down from the reaction through the affinity GST pull down. The samples were then resolved in SDS-PAGE and immunoblotted with anti-HA and anti-SMURF2 antibodies. Note the dramatically increased autoubiquitination of SMURF2WT, but not of its E3 ligase-dead SMURF2*Mut* form, in the presence of peptides: (A) Pep3 and (B) Pep7. The reactions conducted with GST and GST-SMURF2*Mut* proteins show the specificity of the phenomena.

Next, we assessed the ability of the selected SMURF2-targeting compounds, Pep3 and Pep7, to affect the autoubiquitination of SMURF2. To this end, we carried out *in vitro* autoubiquitination assay involving the reconstitution of the ubiquitination reaction in the tube with purified proteins. These include the ubiquitin-activating enzyme E1, ubiquitin-conjugating enzyme E2, HA-tagged ubiquitin, and GST-SMURF2 (either wild-type (SMURF2WT) or E3 ligase-dead Cys716Ala mutant (SMURF2*Mut*)). Purified GST was incorporated into the reaction/s as an additional control. The reactions were conducted in the absence and presence of SMURF2 modifiers used at the concentration range of 1–100 nM. After the incubation and protein pull-down, the samples were extensively washed and resolved in SDS-PAGE, followed by the analysis of SMURF2 autoubiquitination with anti-HA (ubiquitin) antibody. The results (Figure 3(A,B)) showed that addition of either Pep3 or Pep7 to the ubiquitination reaction markedly increased the autoubiquitination levels of SMURF2 at the peptide concentrations of ≥1 nM. Pep7 revealed more profound effect on SMURF2 autoubiquitination in comparison to Pep3. This is in agreement with our other finding showing that Pep7 has lower free binding energy to the HECT domain than Pep3: −48.5 ± 0.4 kcal/mol for Pep7 vs. −38.6 ± 0.3 kcal/mol for Pep3 (Supplementary Figure S1C). The specificity of the measured phenomenon is demonstrated by the absence of SMURF2 autoubiquitination in samples contained either the catalytically inactive SMURF2 or GST (Figure 3, anti-HA/ubiquitin immunoblots: lanes 3, 6, 9, 12 (SMURF2*Mut*) and 1, 4, 7, 10 (GST) vs. lanes 2, 5, 8, 11 (SMURF2WT)).

### 3.4. Cell treatment with Pep7 decreases the protein levels of SMURF2

**Figure 4.**
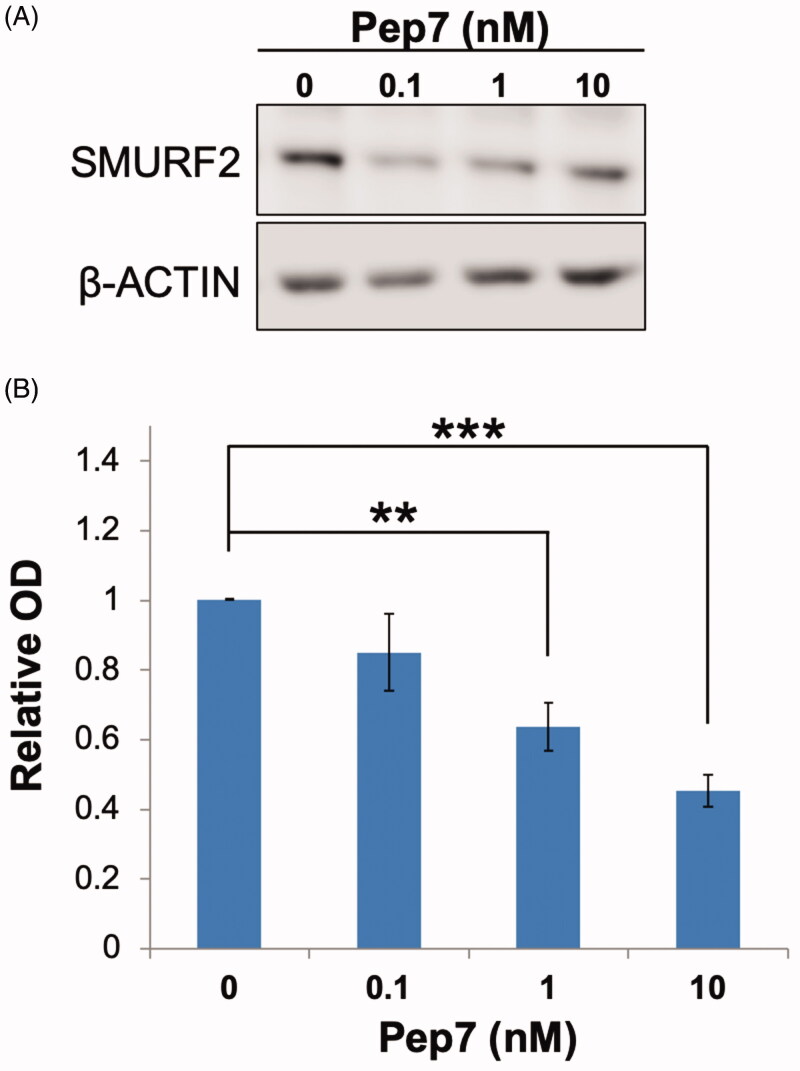
Pep7 significantly decreases SMURF2 protein levels in ANJOU-65 cells. Cells were incubated with different concentrations of Pep7 for 48 h in cultures. SMURF2 expression levels were determined in the total cell extracts using Western blot analysis. (A) Representative immunoblot images showing the effect of Pep7 on the steady-state levels of the endogenous SMURF2. (B) Quantification of the data derived from four independent experiments. Data are mean ± SEM. **p< 0.01; ***p< 0.001.

The ability of E3 ligases to catalyse their own ubiquitination is considered as an important regulatory mechanism governing their expression in the cellular milieu^42–44^. The enhanced autoubiquitination of SMURF2 elicited by Pep7 prompted us to examine whether and how this compound would affect the protein levels of SMURF2 in growing cells. To this end, we treated ANJOU-65 cells (a derivate of human embryonic kidney HEK-293T cells) with different concentrations of Pep7, and assessed its effect on the protein levels of SMURF2 48 hours after the treatment. The results (Figure 4) showed that cell incubation with this compound significantly decreases the steady-state levels of SMURF2 in a dose-dependent manner, which is in line with the results obtained in the *in vitro* studies (Figure 3(A)).

### 3.5. Inactivation of SMURF2 affects cell growth in a context-dependent manner

**Figure 5.**
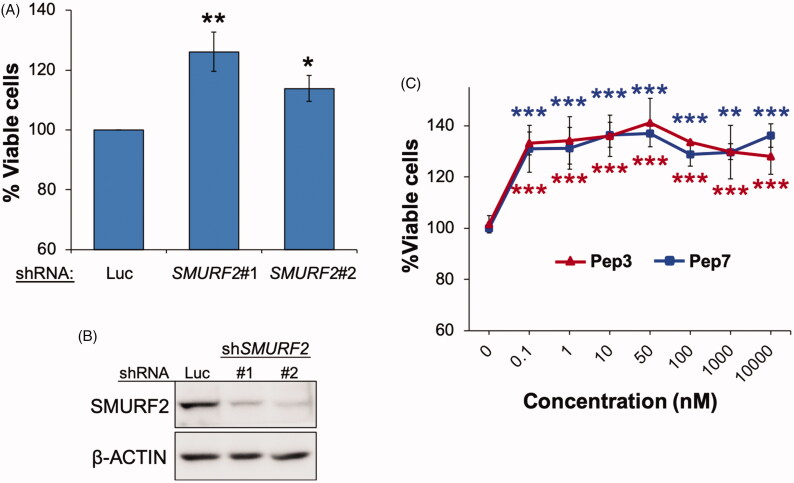
Targeting SMURF2 with either the designated synthetic peptides or with SMURF2-specific shRNA increases cell growth. (A) Effect of *SMURF2* knock-down on proliferation and viability of ANJOU-65 cells measured using a trypan blue exclusion assay. The assay was conducted 72 h after the cell plating. Data are mean ± SEM of four independent experiments. *p< 0.05; **p< 0.01. Two different *SMURF2*-targeting shRNAs were used: sh*SMURF2*#1 (for *SMURF2’s* mRNA coding sequence) and sh*SMURF2*#2 (targeting *SMURF2’s* mRNA at 3′UTR). shRNA against luciferase (shLuc) was used as a control. (B) Western blot analysis showing the effect of *SMURF2*-targeting shRNAs on its expression levels. (C) XTT assay showing the effect of Pep3 and Pep7 on ANJOU-65 cells. Cells were plated at equal density and then incubated with increasing concentrations of the indicated peptides for 72 h. Data are mean ± SD of three-four independent experiments. **p< 0.01; ***p< 0.001.

SMURF2 was previously shown to affect cell proliferation in a cell-context-dependent manner^3^^,^^5^^,^^13^^,^^14^. Our data (Figure 5(A,B)) showed that knock-down of *SMURF2* in ANJOU-65 cells accelerates cell growth, an effect that was consistently monitored with two different shRNAs specific for *SMURF2*. Remarkably, also the treatment of these cells with either Pep3 or Pep7 led to a similar effect and increased cell growth at the nanomolar concentration scale (Figure 5(C)). Noteworthy, in addition to these peptides, increased cell growth was noted also after cell treatment with Pep5, Pep6, Pep9, Pep10, and Pep19, whereas other tested compounds did not show any significant effect (Supplementary Table S1). Furthermore, in addition to ANJOU-65 cells, these compounds were also active in DU-145 prostate carcinoma cell strain, whereas in breast carcinoma MDA-MB-231 cells these molecules showed very little if any effect on cell growth (Supplementary Table S1). One of the possible explanations for this phenomenon could be a cell type specific effect of SMURF2 on cell proliferation. Indeed, neither SMURF2 knockdown nor the gene knockout affected the replication of MDA-MB-231 cells (Supplementary Figure S2A–C), similar to the results obtained in the experiments with SMURF2 protein modifiers (Supplementary Table S1).

### 3.6. Pep7 increases the cytotoxicity of DNA-damaging drug etoposide

**Figure 6.**
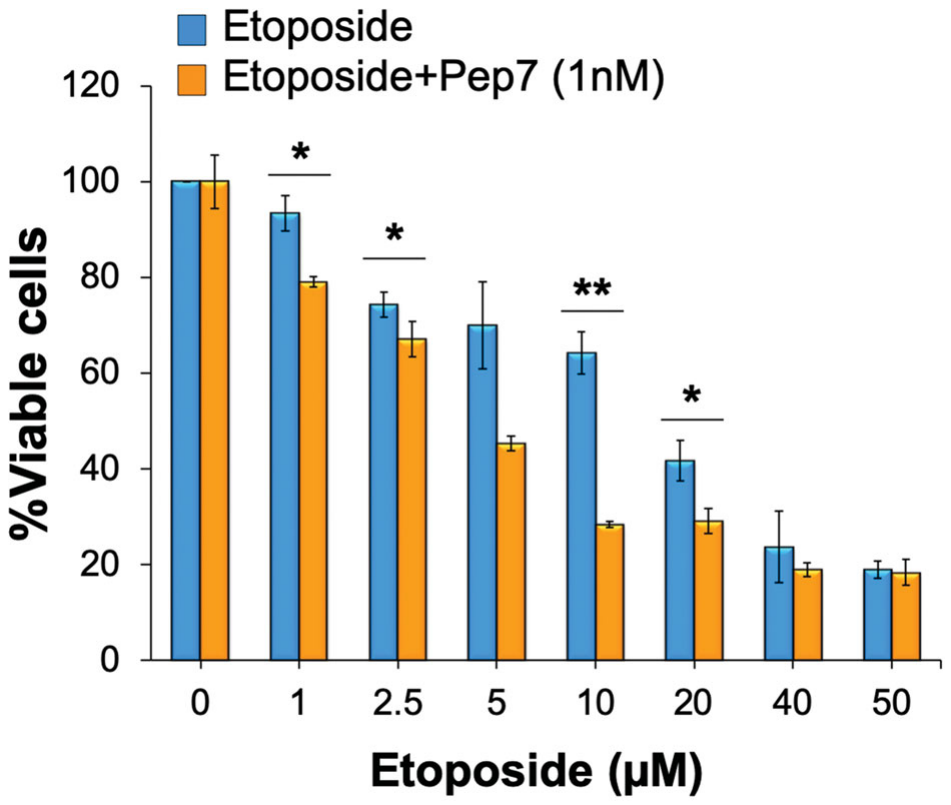
Pep7 affects cell sensitivity to the DNA-damaging drug etoposide. ANJOU-65 cells were treated with either etoposide alone or in combination with 1 nM Pep7 for 72 h. Viable cells were then determined using an XTT assay and compared to untreated cells. Data are mean ± SD of two independent experiments. *p< 0.05; **p< 0.01.

Accelerated replication of certain types of cells following SMURF2 inhibition suggested the possibility that these cells might reveal an increased sensitivity to genotoxic drugs. To test this hypothesis, we treated ANJOU-65 cells with different concentrations of the double-strand-breaks inducer, etoposide, with or without SMURF2 inhibitor Pep7. Seventy-two hours later, the viability of these cells was assessed using XTT assay and compared between etoposide and etoposide-Pep7 treated groups. The results (Figure 6) showed that Pep7 can considerably augment the cytotoxicity of etoposide even at low concentrations of the drug. The data also indicated that the potentiating effect of Pep7 on etoposide-mediated cytotoxicity is pertinent to the ability of SMURF2 to regulate cell growth: as we did not observe any significant difference between the cytotoxicity produced by etoposide treatment in SMURF2-depleted versus SMURF2-proficient MDA-MB-231 cells (Supplementary Figure S2D).

## 4. Conclusions

**Figure 7.**
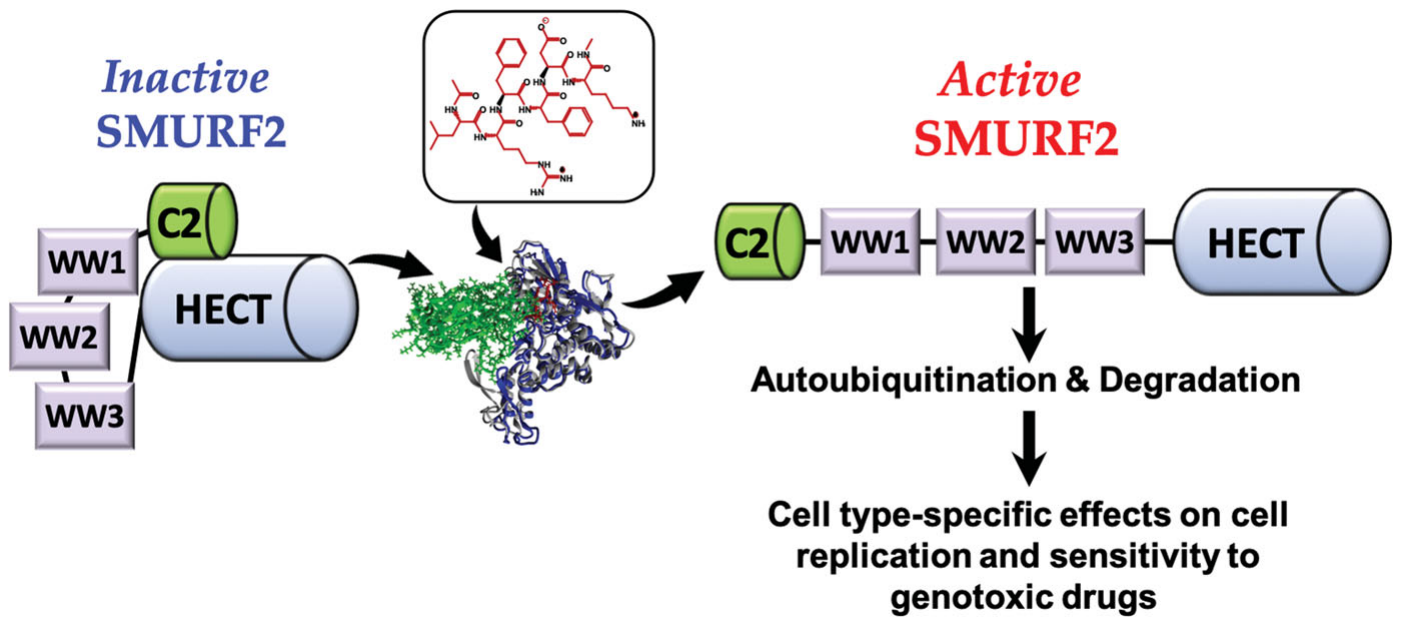
Summary diagram on the activity of SMURF2 targeting modifiers.

The results obtained in our study provide an experimental support to the assumption that the interference with the intramolecular regulation of SMURF2 with short peptides and peptidomimetics can be used to accelerate its autoubiquitination and self-destruction (Figure 7). The data also pointed out that the contextual nature of the effects of SMURF2 modifiers, and their effect on cell sensitivity to genotoxic drugs, are related to the ability of SMURF2 to interfere with cell replication machinery. Future studies, aimed at the design and investigation of SMURF2-targeting small molecules should therefore be conducted in different types of cells: both sensitive and insensitive to the modulation of SMURF2 expression levels and activity.

## Supplementary Material

Supplemental Material
