# Supplementary material for: Development and characterisation of SMURF2-targeting modifiers
Source: J Enzyme Inhib Med Chem. 2021 Jan 12;36(1):401–9. doi: 10.1080/14756366.2020.1871337 (PMC7808752; doi:10.1080/14756366.2020.1871337)
Supplement: Supplemental Material [file IENZ_A_1871337_SM8357.pdf]

Supplementary Material for:

Development and characterization of SMURF2-targeting modifiers

Dhanoop Manikoth Ayyathan, Gal Levy-Cohen, Moran Shubely, Sandy Boutros-Suleiman,

Veronica Lepechkin-Zilbermintz, Michael Shokhen, Amnon Albeck,

Arie Gruzman and Michael Blank

Supplementary Figures S1 and S2

Supplementary Table S1

(A)

Name	Frames	Fraction	Average distance (Å)	+SD
HECT free	25030	1	1.481	0.243
C2	14358	0.987	1.999	0.374
HECT-Pep10	60000	1	1.902	0.445
HECT-Pep7	80000	1	1.869	0.389
HECT-Pep5	95081	0.951	2.458	0.476
HECT-Pep3	84997	1	2.358	0.515

(B)

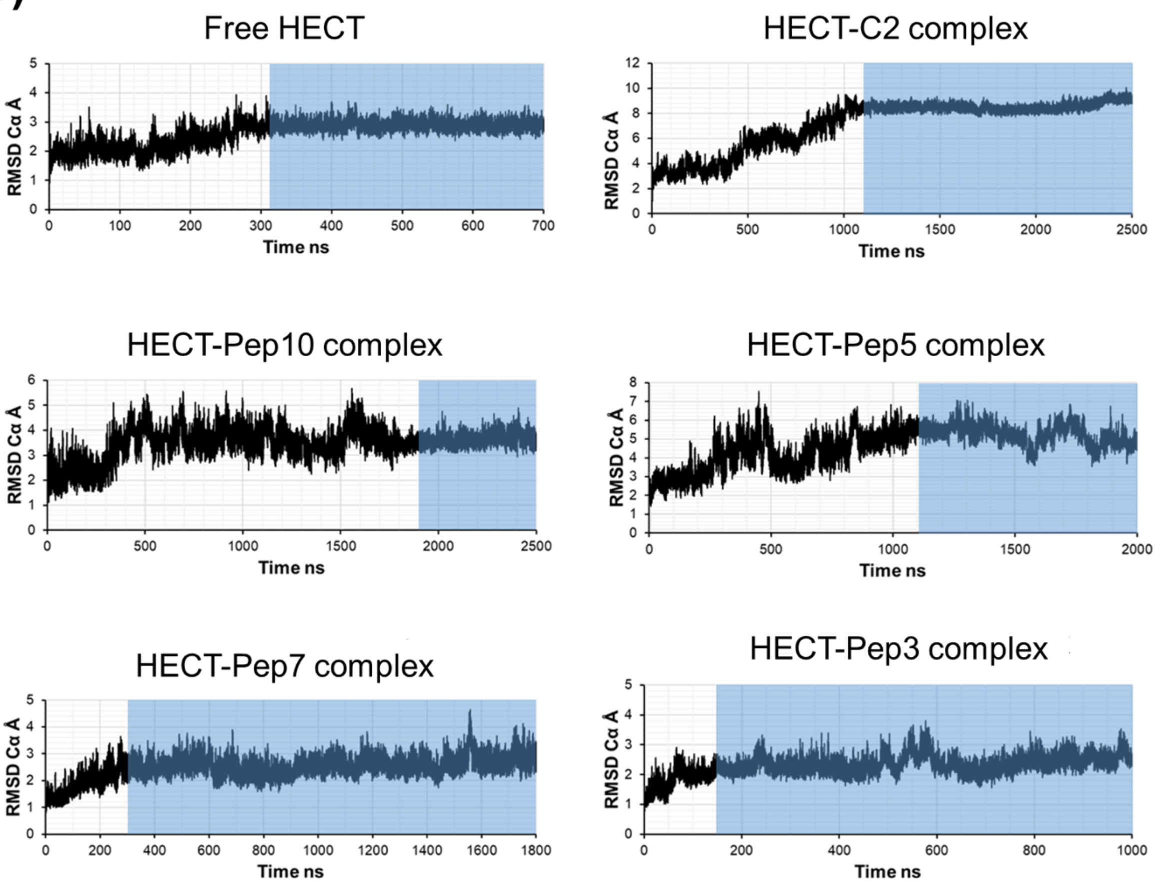

(C)

Peptide inhibitor	Free energy of binding (kcal/mol)	+SD	K_D
C2	-68.5	0.5	5.1E-49
Pep10	-37.5	0.3	3.6E-27
Pep7	-48.5	0.4	6.4E-35
Pep5	-28.1	0.2	1.5E-20
Pep3	-38.6	0.3	6.1E-28

Figure S1. (A) Summary results of conformational cluster centroids of the MD simulated molecular systems calculated by CPPTRAJ software implemented in AMBER16 package. **(B)** RMSD Cα was calculated on the production MD trajectories. The conformational cluster analysis was conducted on the MD trajectory fragment marked by blue rectangular. The considerable RMSD fluctuations in the case of HECT-Pep5 complex are caused by fast conformational exchange between two cluster centroids. **(C)** Calculated free energies of binding of the selected peptide inhibitors to SMURF2 HECT domain and their dissociation constants, K_D.

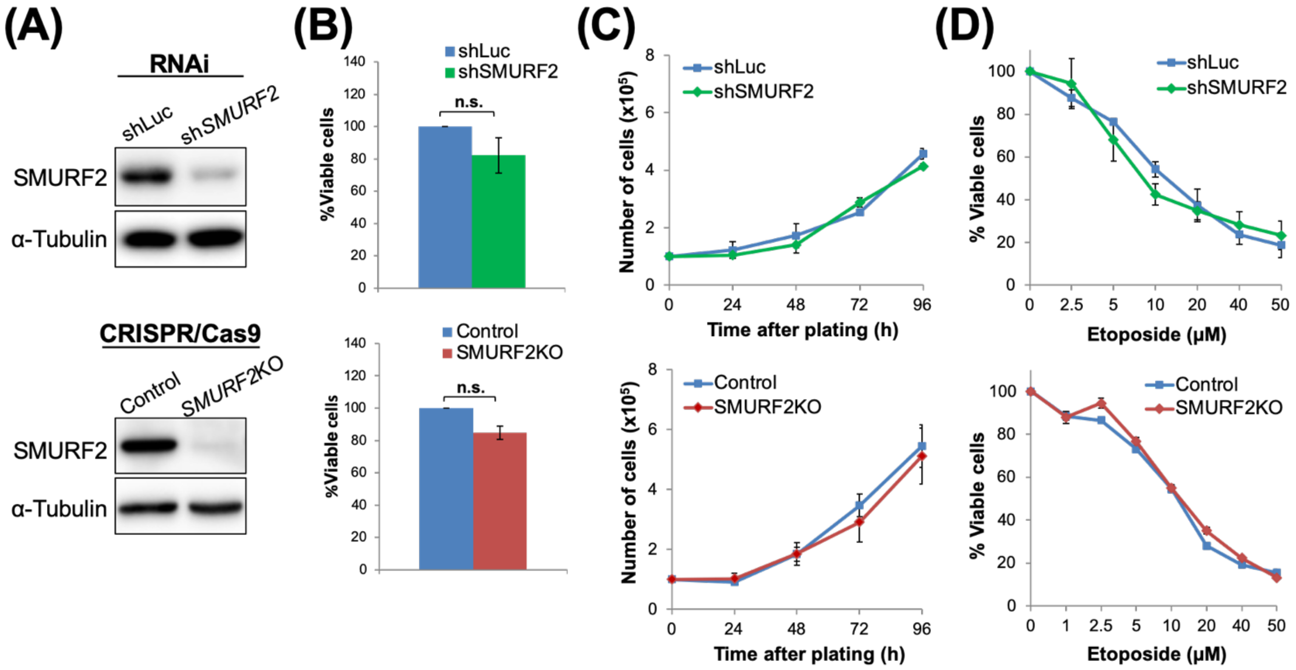

Figure S2. Neither *SMURF2* knockdown nor knockout affect proliferation and sensitivity of MDA-MB-231 cells to etoposide treatment. **(A)** Western blot analysis demonstrating the efficiency of *SMURF2* depletion either through RNAi (shRNA) or CRISPR/Cas9-mediated gene knockout (KO). **(B)** Analysis of growth of *SMURF2*-depleted cells measured by the XTT assay. The data are mean±SEM of four independent experiments performed in triplicates; n.s.–non-significant. **(C)** Analysis of cell proliferation using the trypan blue dye exclusion assay. Data are mean±SEM. Two independent experiments with two technical replicates were performed with *SMURF2*-knockdown cells, and four independent experiments with two technical replicates were conducted with *SMURF2*KO cells. **(D)** XTT assay showing the effect of SMURF2 knockdown and knockout on the sensitivity of MDA-MB-231 cells to etoposide. Data are mean±SEM of three independent experiments performed with three technical triplicates.

Table S1. SMURF2-targeting peptides affect cell growth in peptide- and cell context-dependent manners.

Cell line	Compound	%increase in viable cells relative to controls						
		0.1 nM	1 nM	10 nM	50 nM	100 nM	1000 nM	10000 nM
ANJOU-65	Pep1	3 ± 4.7	31 ± 4.7***	(-1) ± 5.0	3 ± 6.6	25 ± 12.0***	2 ± 9.4	41 ± 13.4***
	Pep2	1 ± 2.0	10 ± 9.3	3 ± 2.9	5 ± 1.9**	14 ± 9.3*	7 ± 9.7	(-6) ± 1.2***
	Pep3	33 ± 4.4***	34 ± 9.2***	36 ± 8.0***	41 ± 9.4***	33 ± 0.2***	29 ± 3.0***	28 ± 7.0***
	Pep4	0 ± 3.6	8 ± 9.8	5 ± 4.8	3 ± 1.0**	27 ± 1.2***	4 ± 4.4	(-4) ± 3.5
	Pep5	38 ± 7.8***	47 ± 7.3***	24 ± 14.09**	46 ± 10.1***	39 ± 25.4**	2 ± 9.08	(-8) ± 13.6
	Pep6	36 ± 10.8 ***	30 ± 2.1***	34 ± 8.3***	39 ± 7.7***	38 ± 7.6***	41 ± 5.1***	33 ± 7.9***
	Pep7	30 ± 9.0***	31 ± 8.1***	36 ± 4.8***	37 ± 0.5***	28 ± 4.6***	29 ± 10.4**	36 ± 4.5***
	Pep8	16 ± 1.9**	12 ± 0.006***	12 ± 2.6*	7 ± 11.06	10 ± 1.8**	6 ± 8.2	3 ± 10.6
	Pep9	20 ± 6.5***	21± 3.4***	18 ± 6.7***	46 ± 6.3***	35 ± 13.5***	(-6) ± 11.1	(-11) ± 13.4
	Pep10	21 ± 10.3**	36 ± 9.1***	28 ± 6.7***	34 ± 6.0***	41 ± 10.8***	36 ± 7.4***	(-17) ± 10.2***
	Pep11	9 ± 1.5***	0 ± 9.8	6 ± 0.1***	3 ± 5.5	6 ± 1.1**	5 ± 9.5	(-8) ± 2.3**
	Pep12	(-1) ± 8.1	1 ± 7.5	(-2) ± 6.4	6 ± 4.1	(-13) ± 2.5	(-7) ± 6.5	(-14) ± 6.0**
	Pep13	13 ± 1.8***	(-5) ± 0.02***	(-9) ± 2.0**	2 ± 3.6	5 ± 6.8	8 ± 2.2**	(-6) ± 3.8
	Pep14	(-8) ± 7.2*	7 ± 14.9	10 ± 6.2*	10 ± 4.7*	(-13) ± 2.5**	1 ± 3.5	(-13) ± 12.2
	Pep15	12 ± 3.0*	14 ± 1.6**	12 ± 1.0**	5 ± 10.6	12 ± 0.7**	8 ± 5.6	6 ± 14.6
	Pep16	12 ± 5.7*	4 ± 0.01***	10 ± 1.4***	3 ± 7.3	6 ± 1.1***	1 ± 4.1	(-1) ± 8.9
	Pep17	(-8) ± 0.9***	(-12) ± 13.2	(-13) ± 4.8**	6 ± 2.5**	(-13) ± 2.3***	(-11) ± 6.5	(-20) ± 4.8**
	Pep18	12 ± 15.2	(-3) ± 8.0	(-13) ± 3.1	12 ± 16.7	6 ± 13.6	(-9) ± 4.2	(-10) ± 1.1***
	Pep19	25 ± 3.6***	17 ± 0.31***	11 ± 5.81*	16 ± 7.3**	33 ± 5.1***	36 ± 5.5***	(-37) ± 2.5***
DU-145	Pep1	2 ± 7.8	4 ± 7.9	6 ± 13.7	3 ± 6.3	8 ± 6.0	9 ± 4.7*	2 ± 4.2
	Pep2	(-1) ± 3.1	4 ± 5.7	5 ± 5.5	5 ± 5.2	5 ± 2.7	6 ± 5.4	6 ± 2.2
	Pep3	15 ± 1.3***	17 ± 5.6***	18 ± 4.8***	19 ± 4.1***	17 ± 6.2***	17 ± 4.0***	7 ± 6.5
	Pep4	3 ± 6.9	4 ± 5.9	1 ± 2.04	5 ± 7.7	2 ± 4.6	6 ± 3.7*	2 ± 3.7
	Pep6	13 ± 5.8**	16 ± 7.1**	15 ± 6.5**	17 ± 1.7***	16 ± 3.9**	15 ± 6.6**	10 ± 5.6*
	Pep7	15 ± 5.2***	12 ± 3.3***	16 ± 6.2***	15 ± 2.1***	18 ± 2.6***	17 ± 1.9***	15 ± 6.06***
	Pep8	(-6) ± 8.07	6 ± 8.9	5 ± 9.5	4 ± 7.8	4 ± 8.0	(-12) ± 7.7	(-2) ± 8.3
	Pep11	1 ± 7.7	0 ± 6.9	0 ± 4.5	2 ± 8.5	0 ± 5.6	(-6) ± 4.6	(-1) ± 10.7
	Pep15	(-1) ± 11.1	4 ± 7.0	3 ± 9.7	4 ± 9.3	(-1) ± 7.5	(-11) ± 7.0	(-1) ± 14.6
	Pep16	9 ± 7.3	6 ± 5.4	5 ± 9.1	4 ± 7.3	4 ± 8.0	4 ± 13.2	(-3) ± 10.0
	Pep19	8 ± 5.1*	6 ± 1.1***	8 ± 2.4**	13 ± 1.04***	15 ± 3.4***	15 ± 0.55***	12 ± 3.8**
MDA-MB-231	Pep1	(-1) ± 2.7	2 ± 5.8	0 ± 5.6	(-6) ± 3.9***	2 ± 9.4	(-2) ± 8.7	(-3) ± 8.0**
	Pep2	1 ± 6.2	1 ± 6.1	(-4) ± 2.6*	(-2) ± 8.8	1 ±12.6	(-7) ± 8.3	(-4) ± 13.4*
	Pep3	10 ± 11.3	11 ± 8.1	5 ± 8.9	8 ± 9.1	6 ± 12.1	(-2) ± 12.7	(-11) ± 12.7
	Pep4	0 ± 6.9	7 ± 1.7***	(-2) ± 2.8	0 ± 4.5	10 ± 9.0	4 ± 3.5	(-9) ± 2.9
	Pep6	4 ± 6.9	6 ± 8.2	4 ± 10.2	4 ± 13.0	3 ± 15.2	(-2) ± 12.7	(-3) ± 15.3
	Pep7	3 ± 9.4	12 ± 10.8	8 ± 10.6	10 ± 7.6**	13 ± 13.8	6 ± 6.8	10 ± 11.1
	Pep8	12 ± 2.04 ***	8 ± 9.8	4 ± 5.1	4 ± 4.6	7 ± 6.2	14 ± 12.05	11 ± 8.21
	Pep11	1 ± 5.3	5 ± 10	7 ± 7.2	5 ± 6.3	4 ± 4.2	19 ± 8.8*	8 ± 12.1
	Pep15	13 ± 1.6**	15 ± 1.9**	4 ± 0.4**	12 ± 2.9*	12 ± 4.5*	20 ± 4.8*	11 ± 9.9
	Pep16	4 ± 9.9	6 ± 7.0	5 ± 6.2	5 ± 6.4	10 ± 3.8**	5 ± 3.3*	(-7) ± 1.6**
	Pep19	10 ± 7.8*	10 ± 7.9*	8 ± 10.7	9 ± 7.6*	11 ± 9.1*	5 ± 4.2*	7 ± 5.5*

Different types of human cell strains were incubated in cultures with the indicated concentrations of examined peptides for 72 hrs. Cell viability was then determined using an XTT assay and compared to related controls (vehicle-treated samples). Values represent the percentage of increase of viable cells in peptide-treated *vs.* vehicle-treated control/s. The data are mean±SD of at least two independent experiments performed in triplicates. $*P < 0.05$; $**P < 0.01$; $***P < 0.001$; $****P < 0.0001$.
